# Dynamic Chromatin Accessibility and Gene Expression Regulation During Maize Leaf Development

**DOI:** 10.3390/genes15121630

**Published:** 2024-12-20

**Authors:** Yiduo Wang, Shuai Wang, Yufeng Wu, Jiawen Cheng, Haiyan Wang

**Affiliations:** National Key Laboratory of Crop Genetics & Germplasm Enhancement and Utilization, Bioinformatics Center, Academy for Advanced Interdisciplinary Studies, Nanjing Agricultural University, Nanjing 210095, China; wangshuai@njau.edu.cn (S.W.); yfwu@njau.edu.cn (Y.W.); jiawen.cheng@higentec.com (J.C.); whaiyan0315@gmail.com (H.W.)

**Keywords:** chromatin accessibility, maize leaf development, ATAC-seq, gene expression regulation, transcription factors

## Abstract

Background/Objectives: Chromatin accessibility is closely associated with transcriptional regulation during maize (*Zea mays*) leaf development. However, its precise role in controlling gene expression at different developmental stages remains poorly understood. This study aimed to investigate the dynamics of chromatin accessibility and its influence on genome-wide gene expression during the BBCH_11, BBCH_13, and BBCH_17 stages of maize leaf development. Methods: Maize leaves were collected at the BBCH_11, BBCH_13, and BBCH_17 developmental stages, and chromatin accessibility was assessed using ATAC-seq. RNA-seq was performed to profile gene expression. Integrated analysis of ATAC-seq and RNA-seq data was conducted to elucidate the relationship between chromatin accessibility and transcriptional regulation. Results: A total of 46,808, 38,242, and 41,084 accessible chromatin regions (ACRs) were identified at the BBCH_11, BBCH_13, and BBCH_17 stages, respectively, with 23.4%, 12.2%, and 21.9% of these regions located near transcription start sites (TSSs). Integrated analyses revealed that both the number and intensity of ACRs significantly influence gene expression levels. Motif analysis identified key transcription factors associated with leaf development and potential transcriptional repressors among genes, showing divergent regulation patterns in ATAC-seq and RNA-seq datasets. Conclusions: These findings demonstrate that chromatin accessibility plays a crucial role in regulating the spatial and temporal expression of key genes during maize leaf development by modulating transcription factor binding. This study provides novel insights into the regulatory mechanisms underlying maize leaf development, contributing to a deeper understanding of chromatin-mediated gene expression.

## 1. Introduction

Maize (*Z. mays*) is one of the world’s most significant staple crops and serves as a valuable model organism in plant science due to its well-characterized genome and rapid growth [1]. Maize leaves, as the primary sites of photosynthesis, are critical in determining crop yields, highlighting the importance of studying leaf development in plant biology [2]. Leaf development in maize is a tightly regulated process that significantly impacts plant productivity and resilience to environmental stresses. Consequently, understanding the molecular mechanisms governing maize leaf development is crucial for enhancing crop performance under diverse environmental conditions.

The BBCH scale, a decimal coding system representing phenological stages in monocotyledonous and dicotyledonous species, was developed by the Biologische Bundesanstalt, Bundessortenamt, and Chemical industry [3]. This study primarily focused on the leaf development stages within Principal Growth Stage 1, specifically BBCH_11 (first leaf unfolded), BBCH_13 (three leaves unfolded), and BBCH_17 (seven leaves unfolded).

At the BBCH_11 stage, maize leaves are in the early developmental phase, relying primarily on seed reserves for energy, while the meristem remains underground. During the BBCH_13 stage, the meristem stays below ground, but the plant transitions to photosynthesis as its primary energy source. The BBCH_17 stage marks rapid leaf growth, with the meristem emerging above ground and the plant becoming increasingly sensitive to environmental stresses, including drought and nutrient limitations. These developmental stages are pivotal for understanding the dynamic changes in chromatin accessibility, a key mechanism regulating gene expression, during maize leaf development.

The growth of maize leaves is regulated by various genetic, transcriptional, and post-transcriptional mechanisms [4,5]. Transcription factors (TFs), essential regulatory proteins that bind to specific DNA sequences through their DNA-binding domains, play a critical role in controlling leaf development by either promoting or repressing mRNA transcription [6]. Two key aspects of leaf development, chlorophyll biosynthesis and leaf angle, are influenced by TFs. For example, *GLKs* and *MYBR26* are vital modulators of chlorophyll biosynthesis. Mutations in *GLK* genes disrupt the expression of essential photosynthesis-related genes, significantly impacting maize leaf formation and function [7,8]. Although the role of *MYBR26* in maize remains poorly understood, its Arabidopsis homologs are associated with circadian regulation, suggesting a similar regulatory function in maize leaf development [9]. Another important group of TFs, the CONSTANS-LIKE proteins, regulate photosynthesis by modulating light-responsive gene expression, thereby optimizing photosynthetic efficiency during leaf growth [10]. In addition, *ZmNF-YC13* influences leaf angle by regulating *ZmWRKY76* and *ZmBT2* expression in coordination with *ZmNF-YBs* and *ZmNF-YA3* [11]. Certain TFs, known as pioneer factors, can enhance chromatin accessibility by recruiting chromatin-remodeling complexes or displacing nucleosomes [12]. Research involving 3D genome assembly and quantitative trait locus (QTL) analysis has shown that changes in chromatin accessibility can affect the long-term regulatory functions of TFs [13]. Therefore, understanding the interaction between TFs and chromatin accessibility is critical for elucidating the mechanisms underlying maize leaf development.

In eukaryotes, nuclear DNA is organized into a repetitive nucleosomal structure, where DNA is wrapped around histone octamers. The binding of regulatory proteins to active regulatory elements often necessitates nucleosome displacement or removal to create accessible regions for protein interactions [14]. Consequently, chromatin accessibility is a critical indicator of how structural modifications in chromatin regulate gene transcription. TFs, for example, can activate transcription by disrupting nucleosome assembly at regulatory regions, such as enhancer, promoter, insulator, and locus control regions. These regulatory DNA sequences are pivotal in modulating chromatin accessibility, directly impacting gene expression [14].

Several techniques, including formaldehyde-assisted isolation of regulatory elements sequencing (FAIRE-seq), assay for transposase-accessible chromatin using high-throughput sequencing (ATAC-seq), and DNase I hypersensitive site sequencing (DNase-seq), have been developed to profile chromatin accessibility [15]. ATAC-seq is favored among these because of its many benefits: it has been extensively used in numerous studies, uses fewer cells, and requires less time to prepare samples [16]. Although recent research has utilized ATAC-seq to investigate chromatin accessibility in tissues such as the root, embryo, and endosperm, the chromatin accessibility landscape in maize leaves during the BBCH_11, BBCH_13, and BBCH_17 stages remains largely unexplored [17,18,19].

This study investigated the dynamics of chromatin accessibility and transcriptome profiles in B73 maize leaves at the BBCH_11, BBCH_13, and BBCH_17 developmental stages using ATAC-seq and RNA-seq. These findings enhance our understanding of gene regulation during maize leaf development and provide a theoretical foundation for exploring the molecular mechanisms underlying leaf growth across different developmental stages.

## 2. Materials and Methods

### 2.1. Sample Description

The maize (*Z. mays*) samples used in this study were obtained from the experimental fields at Henan Agricultural University. Sampling was performed at three developmental stages: BBCH_11, BBCH_13, and BBCH_17, corresponding to the fully expanded first, third, and seventh leaves, respectively. For each stage, the innermost leaves were harvested. Two biological replicates were collected per developmental stage, and the tissues were immediately flash-frozen in liquid nitrogen for storage.

### 2.2. ATAC-Seq Library Preparation

A total of six samples were prepared for ATAC-seq library construction. Approximately 50,000 freshly isolated cells were obtained from each sample. After a series of centrifugation steps, a 50 μL tagmentation reaction mix was added, consisting of 25 μL of 2× Nextera reaction buffer, 2.5 μL of Nextera Tn5 transposase, and 22.5 μL of nuclease-free water. The reaction mixture was incubated at 37 °C for 30 min, followed by PCR amplification for eight cycles. The resulting libraries were purified using the MinElute PCR Purification Kit (Qiagen, Hilden, Germany), and their quality was assessed with an Agilent Bioanalyzer 2000 (Agilent, Santa Clara, CA, USA). Sequencing was conducted using 150 bp paired-end reads on a NovaSeq 6000 system (Illumina, San Diego, CA, USA).

### 2.3. RNA-Seq Library Preparation

Total RNA was isolated from all samples utilizing the TruSeq Stranded Total RNA Ribo-Zero H/M/R Kit (Illumina RS-122-2201). The integrity of the RNA was assessed through electrophoresis on a 1.5% agarose gel, while its purity and concentration were determined with a NanoDrop spectrophotometer. RNA samples meeting high-quality standards were subsequently used to construct RNA-seq libraries, which were sequenced as 150 bp paired-end reads on the HiSeq X system.

### 2.4. ATAC-Seq Data Analysis

Raw sequencing fragments were preprocessed using trim_galore-0.4.4 to remove sequences containing adapter contaminants [20]. After trimming, FastQC-0.11.5 was employed to assess the quality of the resulting fragments. Reads were then aligned to the B73 RefGen_v4 reference genome using Bowtie2-2.2.5 (https://bowtie-bio.sourceforge.net/bowtie2, 16 November 2024) with default settings [21]. Fragments with mapping quality scores below 30 or those aligning to chloroplast or mitochondrial DNA were filtered out using SAMtools-1.8 [22]. Accessible chromatin regions (ACRs) were identified through MACS 2.0, applying a q-value threshold of 0.05 [23]. To generate representative ACR sets for each developmental stage, shared ACRs were merged using BEDTools-2.26.0 [24]. For the visualization of genome-wide chromatin accessibility peaks in the Integrative Genomics Viewer (IGV) and for analyzing signal patterns near gene bodies, BAM files were converted to BigWig format using DeepTools-3.5.1 [25]. Promoter peaks were defined as those located between −2 kb and 0 bp relative to transcription start sites (TSSs).

To assess peak enrichment within various genomic regions, random regions were generated using BEDTools-2.26.0 and annotated with custom scripts. This procedure was repeated 5000 times, and the average annotation values obtained for each region served as random controls. The relationship between the peak density and chromosome length was analyzed by normalizing chromosome length (normalized chromatin length = total peak count × chromatin length/total genome length), followed by calculating the Pearson correlation coefficient to evaluate the association between the normalized chromatin length and peak numbers. Distinct and shared peaks across developmental stages were determined with the help of BEDTools-2.26.0 [24].

To explore the association between peak length and gene expression, genes possessing a single ACR within their promoter regions were sorted by ACR length and categorized into three groups of equal size (top, middle, and bottom). When ACRs exhibited identical lengths, they were allocated to groups aiming to balance the distribution of similar-length ACRs. Differential peak intensity (DPI) across developmental stages was analyzed using DESeq2-2.13, where significant DPI was determined based on an adjusted *p*-value < 0.05 and an absolute log2-fold change exceeding 1 [26]. Enrichment analyses for Gene Ontology (GO) term pathways were carried out using the R package topGO-2.40.0 [27], with a *p*-value threshold of 0.05 considered significant. To identify cis-regulatory elements (CREs), motif analysis of the peaks was conducted with HOMER’s findMotifsGenome function, employing default parameters [28], and motifs with a *p*-value below 0.01 were considered noteworthy.

### 2.5. RNA-Seq Data Processing

For each sample, the raw sequencing reads were preprocessed with trim_galore-0.4.4 to eliminate adapter sequences and bases of low quality [20]. The cleaned reads were then mapped to the B73 RefGen_v4 reference genome using HISAT2-2.1.0 [29], with unmapped reads being filtered out using SAMtools-1.8 [22]. Exonic read counts were calculated using HTSeq-2.0.3 [30]. Differentially expressed genes (DEGs) were identified following the same criteria applied for ATAC-seq, and gene expression levels were quantified as fragments per kilobase per million mapped reads (FPKM) using StringTie-1.3.6 [31]. Subsequent analyses focused on genes with FPKM values exceeding 1 in at least three samples at a single stage.

## 3. Results

### 3.1. Genome-Wide Identification of ACRs in Maize Leaf Development

**Figure 1:**
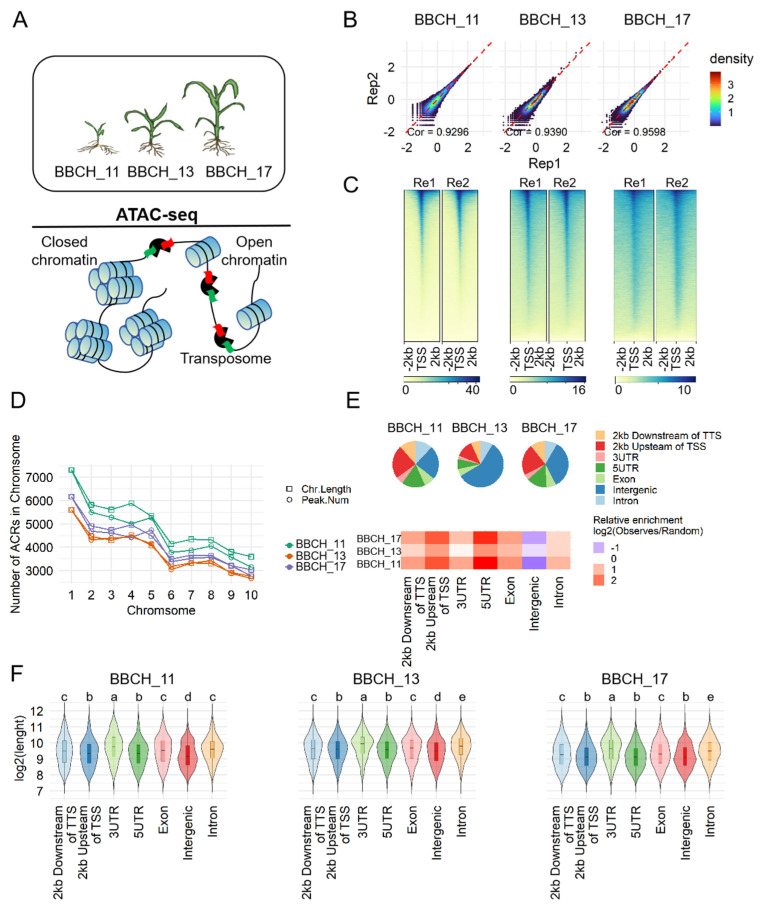
The experimental setup and ATAC-seq quality assessment. (**A**) A schematic of leaf tissue collection from maize at the BBCH_11, BBCH_13, and BBCH_17 developmental stages for ATAC-seq analysis, where Tn5 transposase preferentially cleaves DNA at accessible sites while inserting sequencing adapters. (**B**) Scatterplots illustrating the Pearson correlations of normalized genome-wide ATAC-seq signals (reads per million, RPM) between biological replicates at the BBCH_11, BBCH_13, and BBCH_17 stages. (**C**) Heatmaps depicting the normalized ATAC-seq signals at all transcription start sites (TSSs), organized by signal intensity. (**D**) The distribution of accessible chromatin region (ACR) counts across different chromosomes. (**E**) Top: Proportions of ACRs in various genomic regions. Bottom: Fold enrichment of ACRs in different genomic regions. (**F**) A summary of ACR lengths in distinct genomic regions. Violin plots illustrate the distribution of ACR lengths at the BBCH_11, BBCH_13, and BBCH_17 stages. Statistical differences between groups are indicated by different letters above the violins, determined by Tukey’s honest significant difference test (*p* < 0.05).

We performed a genome-wide investigation of accessible chromatin regions (ACRs) associated with maize leaf development by profiling chromatin accessibility at the BBCH_11, BBCH_13, and BBCH_17 developmental stages using ATAC-seq (Figure 1A). The ATAC-seq analysis included six biological samples, generating an average of 86 million uniquely mapped reads per sample aligned to the reference genome (Table S1). Library quality was assessed by examining the biological replicates’ correlations and the distribution of Tn5 integration signals around transcription start sites (TSSs). Correlation coefficients among biological replicates exceeded 0.92, with Tn5 signals showing peak intensity at TSSs (Figure 1B,C). This analysis identified numerous high-confidence ACRs across the six libraries (Table S1), with fewer ACRs detected in older leaves compared to younger leaves, suggesting a decline in chromatin accessibility as leaves mature. To determine the most representative accessible regions, we analyzed the overlap of ACRs between replicates at each stage. We identified 46,808, 38,242, and 41,084 ACRs at the BBCH_11, BBCH_13, and BBCH_17 stages, respectively, representing 4.4%, 5.1%, and 3.4% of the maize genome. These ACRs were used for all subsequent analyses.

We investigated the correlation between the number of ACRs and chromosome length, finding that chromosomes with greater length tended to have a higher density of ACRs (r^2^ = 0.982 for BBCH_11, r^2^ = 0.995 for BBCH_13, r^2^ = 0.980 for BBCH_17) (Figure 1D). To gain deeper insights into ACR distribution across the genome, we examined their positions relative to the overall genomic structure (Figure 1E). At each developmental stage, ACRs were predominantly located in intronic and intergenic regions, which comprised approximately 35.6% to 66.4% of all identified peaks. ACRs within promoter regions (2 kb upstream of TSS) accounted for approximately 23.4%, 12.2%, and 21.9% of total peaks at the BBCH_11, BBCH_13, and BBCH_17 stages, respectively. ACRs were less frequently observed in 3’ UTR and exonic regions. Overall, ACRs were more enriched in promoter and 5’ UTR regions compared to intergenic regions (Figure 1E).

Furthermore, we assessed the mean lengths of ACRs across various genomic regions (Figure 1F). ACRs in the 3’ UTR regions were found to be longer on average compared to those in other genomic regions, while the shortest ACRs were detected in intergenic areas.

### 3.2. Regulation of Gene Expression Through Coordination by ACRs

RNA-seq was conducted on the same developmental stages as used for ATAC-seq to assess how ACRs influence gene expression. The average unique sequence alignment across all samples reached 90% (Table S2). Correlation analysis revealed a high correlation coefficient (above 0.98) between biological replicates, indicating strong experimental repeatability (Figure S1). To investigate the regulatory influence of chromatin accessibility on gene expression, we concentrated on ACRs annotated to promoter regions, which showed notable enrichment (Figure 1E).

**Figure 2:**
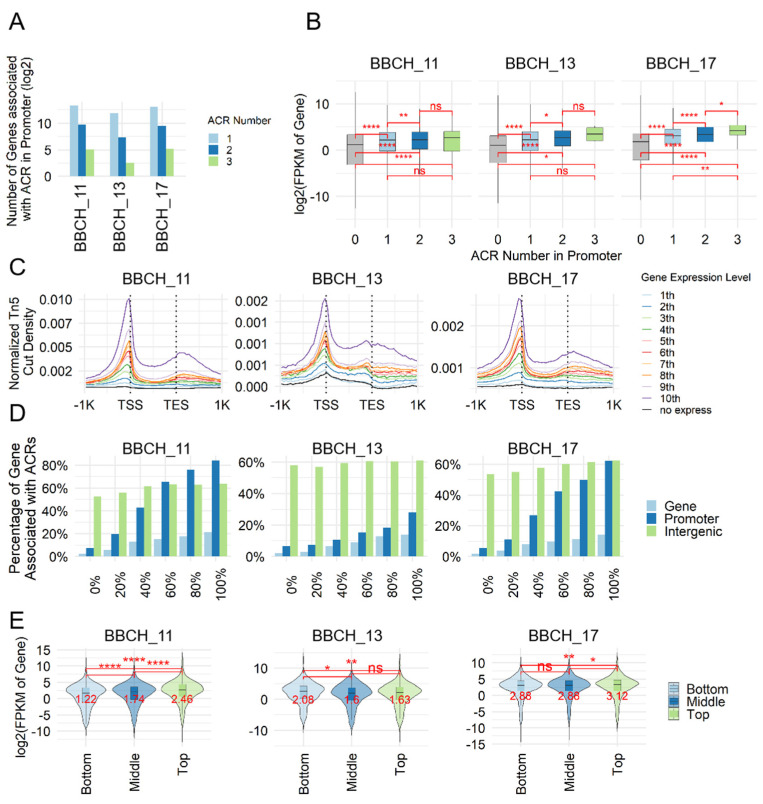
Gene expression levels associated with ACRs. (**A**) The distribution of ACR numbers in the promoter regions of genes. (**B**) Boxplots displaying gene expression levels associated with ACRs in different groups. The labels “0~3” represent groups of genes containing 0 to 3 ACRs in their promoter regions, respectively. Statistical significance was determined using the Wilcoxon signed-rank test, with significance levels indicated as * < 0.05; ** < 0.01; **** < 0.0001; ns: not statistically significant. (**C**) The profile of Tn5 sensitivity (measured by ATAC-seq read counts) across genes with different expression levels. Genes were sorted into 10 bins based on their expression levels, from low expression (first bin) to high expression (10th bin). (**D**) The proportion of genes with varying expression levels (based on RNA-seq data, FPKM) associated with ACRs in different genomic regions. Genes were categorized into six groups based on expression levels: no expression (FPKM = 0) and five additional bins from the lowest 20% to the highest 20% (labeled as 100% in the figure). (**E**) Boxplots showing the association of gene expression levels with ACRs in three equal groups (top, middle, and bottom) based on ACR peak length. Statistical significance was assessed using the Wilcoxon signed-rank test, with significance levels indicated as * < 0.05; ** < 0.01; **** < 0.0001; ns: not statistically significant. Median values are shown in the figure.

Our analysis revealed that more than 92% of the genes linked to these ACRs harbored a single ACR within their promoter regions (Figure 2A). Next, we examined how the number of ACRs in the promoter correlates with gene expression levels. At all developmental stages, genes associated with ACRs demonstrated higher expression levels compared to those without ACRs. Additionally, genes with more than one ACR in the promoter exhibited higher expression levels than genes with only one ACR (Figure 2B).

To understand the relationship between gene expression and chromatin accessibility upstream and downstream of genes, we categorized genes based on their expression levels and assessed the accessibility within 2 kb upstream and downstream regions. Our results indicated that genes with high expression levels were linked to enhanced accessibility at both the transcription start site (TSS) and the transcription end site (TES) (Figure 2C). Additionally, more than 60% of the top 20% most highly expressed genes in BBCH_11 and BBCH_17 were associated with ACRs located within both the gene body and promoter areas (Figure 2D).

We then classified ACRs into three categories (top, middle, and bottom) based on their length and analyzed their relationship with gene expression levels. Except for the BBCH_13 stage, genes linked to the top ACR group showed higher expression levels than those associated with the bottom ACR group (Figure 2E). These results suggest that genes with longer ACRs are likely to have higher expression levels.

### 3.3. Developmentally Specific and Differential ACRs During Leaf Growth

**Figure 3:**
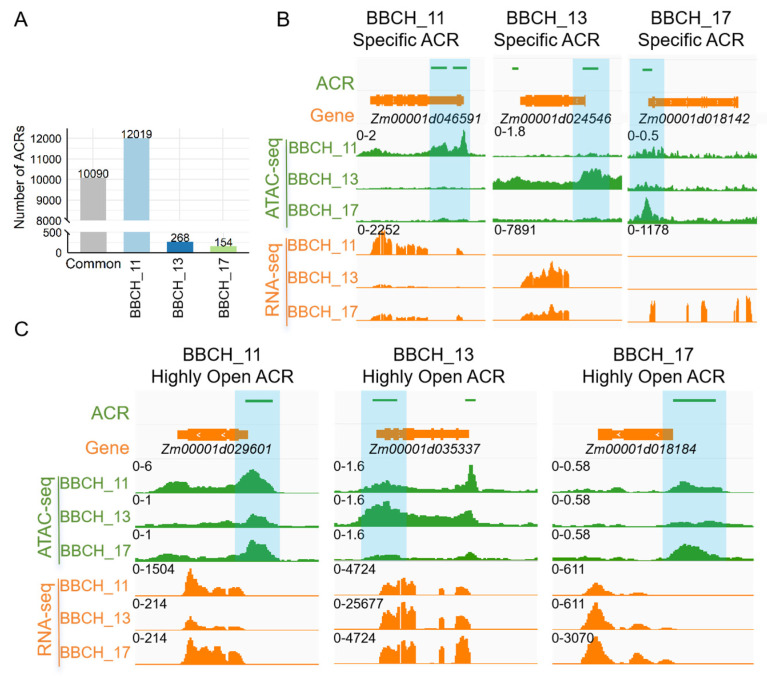
ACR changes throughout leaf development. (**A**) Quantification of stage-common and stage-specific ACRs across the BBCH_11, BBCH_13, and BBCH_17 stages. (**B**) Visualization of genes associated with stage-specific ACRs across different developmental stages. (**C**) Visualization of genes associated with stage-common ACRs across different developmental stages.

A substantial number of ACRs were identified at all developmental stages investigated in this study; however, distinct differences were evident when comparing genome-wide ACRs across these stages. Specifically, 12,019, 268, and 154 stage-specific ACRs were identified at the BBCH_11, BBCH_13, and BBCH_17 stages, respectively. For example, the gene *Zm00001d046591*, associated with leaf development, had a stage-specific ACR in its promoter region that was present only at the BBCH_11 stage (Figure 3B). In contrast, *Zm00001d024546* (LHY: LATE ELONGATED HYPOCOTYL), a gene responsive to gibberellin signaling and highly expressed in differentiating leaves [32], exhibited a BBCH_13-specific ACR in the promoter region (Figure 3B). Furthermore, *Zm00001d018142*, associated with leaf morphogenesis, exhibited a BBCH_17-specific ACR (Figure 3B). These results suggest that ACRs exhibit dynamic variation during leaf development. The alterations in ACRs at each stage were aligned with the expression levels of the corresponding genes, as illustrated in Figure 3B. The peak expression levels for *Zm00001d046591* and *Zm00001d024546* were observed at the BBCH_11 and BBCH_13 stages, respectively, while the highest expression of *Zm00001d018142* was detected at the BBCH_17 stage.

We then examined the expression patterns of genes associated with stage-specific ACRs across all stages (Figure S2A). The analysis revealed that a significant number of these genes (1870) had elevated expression at their corresponding stages. In contrast, 2320 genes did not show increased expression during their active stage. Taken together, our results imply that ACRs might influence gene expression, at least in part, by altering the binding sites for transcription factors (TFs).

Additionally, we identified 10,090 common ACRs present across all three leaf stages (Figure 3A). Interestingly, the intensities of certain ACRs varied across the different stages. For instance, within the coding region and promoter areas of *Zm00001d029601*, a gene involved in simple leaf morphogenesis, chromatin accessibility decreased from BBCH_11 to BBCH_17, with ACR intensity at BBCH_11 being greater than at the later stages (Figure 3C). In parallel, *Zm00001d029601* exhibited the highest expression level at the BBCH_11 stage (Figure 3C).

We performed differential peak intensity (DPI) analysis on the common ACRs across the different stages (Table 1). Comparisons of BBCH_11 vs. BBCH_13, BBCH_11 vs. BBCH_17, and BBCH_13 vs. BBCH_17 identified 2619, 1775, and 714 ACRs, respectively, with differential peak intensities (DPIs). Correlation analysis between peak intensity and the expression levels of the corresponding genes revealed that over 75% of the genes showed a positive correlation, with the median correlation coefficient surpassing 0.75 (Figure S2B). These findings suggest that variations in ACR intensity associated with these genes could play a significant role in modulating their expression.

Furthermore, as shown in Table 1 and Figure 3A, a higher number of peaks with significant intensity differences were observed in the BBCH_11 vs. BBCH_13/BBCH_17 comparisons or among stage-specific ACRs at the BBCH_11 stage. This suggests that substantial changes in chromatin accessibility take place during leaf development, especially between the BBCH_11 and BBCH_13/BBCH_17 stages.

### 3.4. Stage-Specific Regulation of Genes During Leaf Development

**Figure 4:**
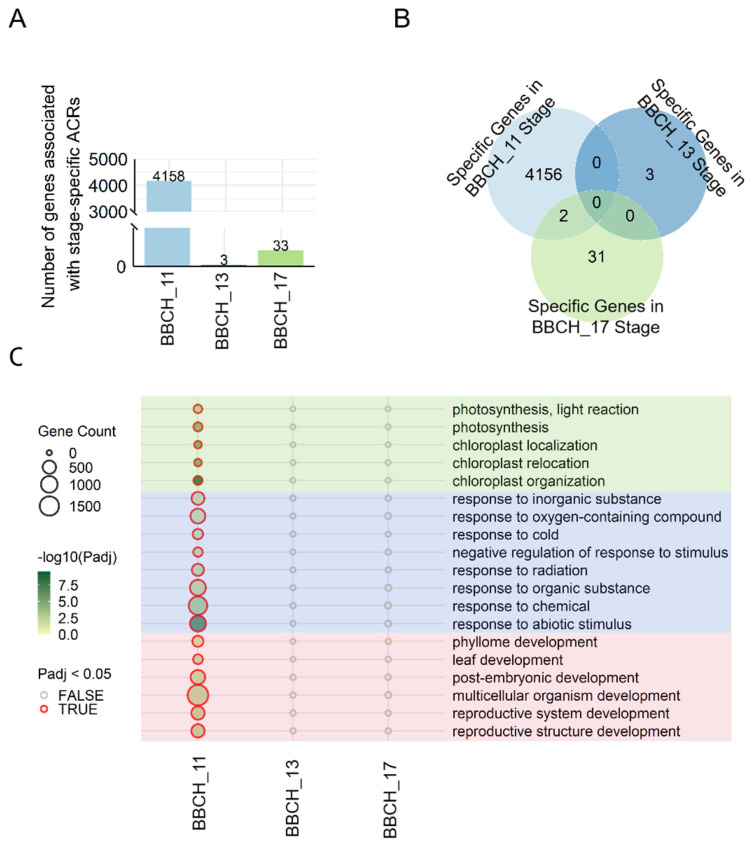
Functional analysis of genes linked to stage-specific ACRs. (**A**) The number of genes associated with stage-specific ACRs. (**B**) Counts of shared and stage-specific genes with ACRs at the BBCH_11, BBCH_13, and BBCH_17 stages. (**C**) Enriched biological processes (BPs) in the BBCH_11, BBCH_13, and BBCH_17 groups. The dot color indicates the statistical significance (*p*-value), and the dot size reflects the number of genes associated with the respective terms.

To further explore the regulatory functions of genes associated with stage-specific ACRs and differential peak intensities (DPIs) during leaf development, we analyzed the ACR-related genes and their biological roles. We identified 4158, 3, and 33 genes with stage-specific ACRs at the BBCH_11, BBCH_13, and BBCH_17 stages, respectively (Figure 4A), showing limited overlap between these gene sets (Figure 4B). Gene Ontology (GO) analysis was performed to determine the biological functions of these stage-specific ACR-related genes. The analysis showed notable enrichment of these genes in biological processes (BPs) related to leaf development (Table S3 and Figure 4C). Specifically, genes associated with BBCH_11-specific ACRs were enriched in processes such as photosynthesis, chloroplast organization, response to abiotic stress, and reproductive development. In contrast, genes with stage-specific ACRs at BBCH_13 and BBCH_17 did not exhibit significant enrichment in any particular biological processes.

**Figure 5:**
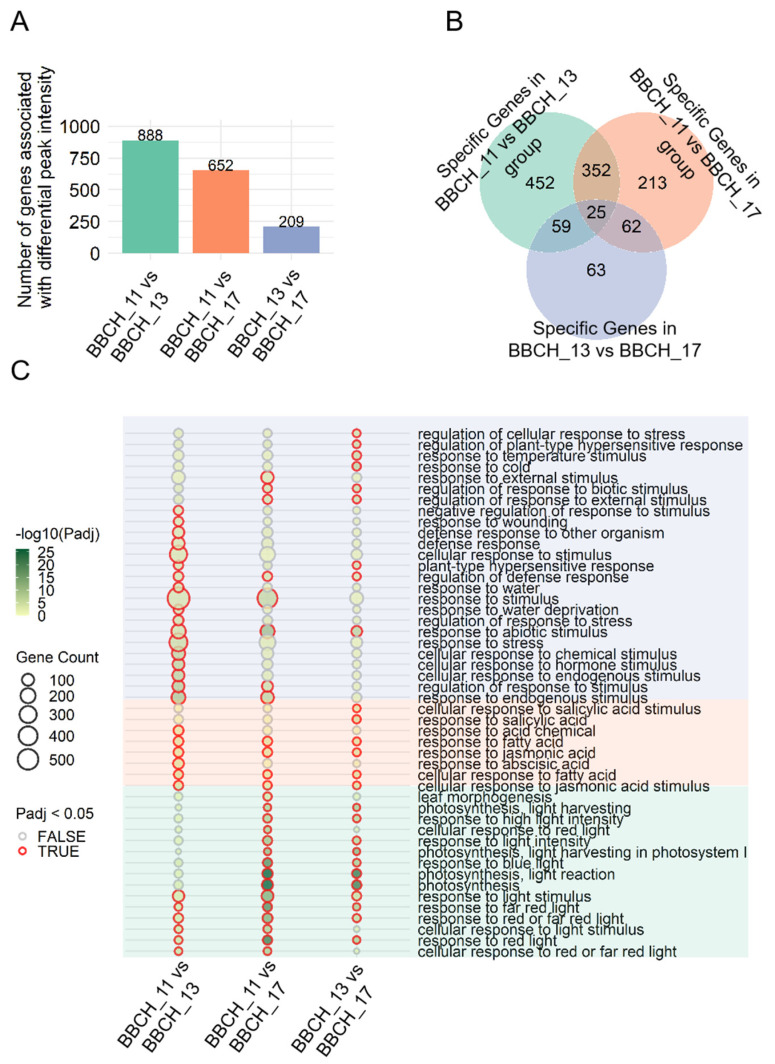
Functional analysis of genes linked to differential peak intensities (DPIs). (**A**) The number of genes with DPIs in ACRs common to all developmental stages. (**B**) Counts of shared and stage-specific genes linked to DPIs from the BBCH_11 vs. BBCH_13, BBCH_11 vs. BBCH_17, and BBCH_13 vs. BBCH_17 comparisons. (**C**) Enriched biological processes (BPs) related to photosynthesis, hormones, and stress responses in the BBCH_11 vs. BBCH_13, BBCH_11 vs. BBCH_17, and BBCH_13 vs. BBCH_17 groups. The color of the dots reflects the statistical significance (*p*-value), while the dot size corresponds to the number of genes enriched for each term.

To analyze the common ACRs, we first mapped the identified DPIs to the genome and then conducted functional analyses based on the annotated genes. From each comparison, we identified 888, 652, and 209 genes, respectively (Figure 5A). Similar to the genes associated with stage-specific ACRs, the genes identified by DPIs in each comparison were significantly enriched in several leaf-related biological processes (BPs) (Tables S4–S6 and Figure 5C), including response to light and photosynthesis. Notably, terms identified in the BBCH_11 vs. BBCH_17 comparison were significantly associated with leaf morphogenesis (Figure 5C). Several stress-related pathways, including the response to water and cold, as well as the response to wounding, were also significantly enriched among the genes displaying DPIs in these comparisons. Moreover, hormone-related pathways, which are essential for leaf development, including the response to jasmonic acid and abscisic acid, were notably enriched among the genes identified in all three comparisons.

We identified 25 genes with DPIs that were common to all three comparisons (Figure 5B). Notably, *Zm00001d035859* and *Zm00001d042673* were correlated with leaf morphogenesis, while gene *Zm00001d038163* was associated with photosynthesis. Furthermore, genes *Zm00001d005911*, *Zm00001d035859*, *Zm00001d038163*, and *Zm00001d049387* were linked to the response to light.

### 3.5. Identification of Regulatory DNA Elements Across Various Stages of Leaf Development

**Figure 6:**
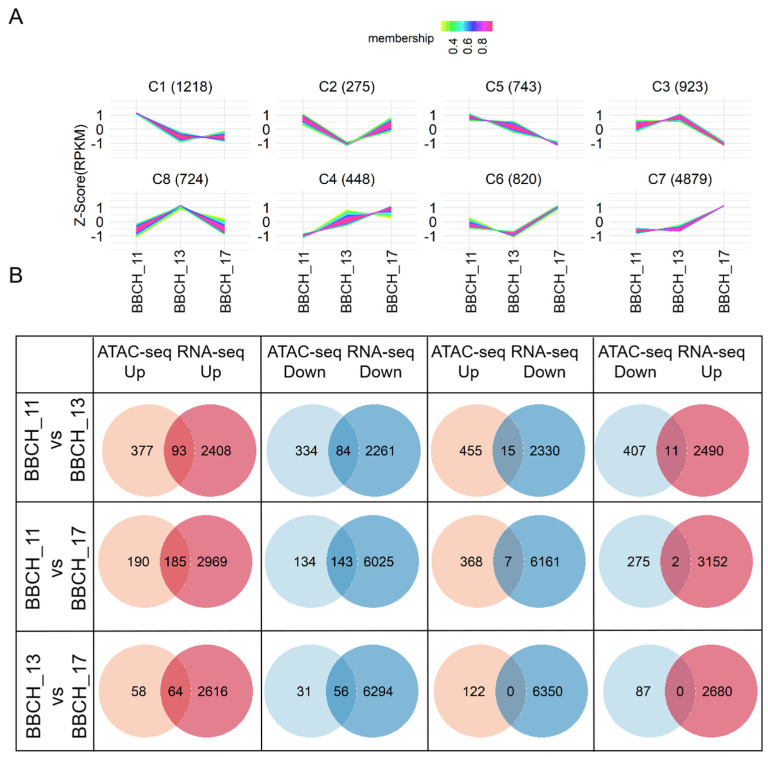
Identification of regulatory DNA elements across different stages of leaf development. (**A**) Expression profiles of all differentially expressed genes (DEGs) categorized into eight clusters based on their expression patterns. (**B**) The intersection of DPI-related genes identified by ATAC-seq with differentially expressed genes (DEGs) detected through RNA-seq. “ATAC-seq down” refers to DPI-related genes that were downregulated in ATAC-seq; “ATAC-seq up” refers to DPI-related genes that were upregulated in ATAC-seq; “RNA-seq down” refers to DEGs that were downregulated in RNA-seq; and “RNA-seq up” refers to DEGs that were upregulated in RNA-seq.

The gene expression profiles showed marked variation across different stages of leaf development. By performing pairwise differential expression analysis across the different stages, we identified 10,031 genes with differential expression (DEGs). The identified DEGs were grouped into eight clusters (C1~C8) according to their expression patterns (Figure 6A). Genes in C1 and C2 displayed specific expression during BBCH_11; genes in C8 were specifically expressed during the BBCH_13 period; genes in C6 and C7 were specifically expressed in the BBCH_17 period; the expression of genes in C3 and C5 was upregulated in periods other than the BBCH_17 period; and the expression of genes in C4 was upregulated in all periods except for the BBCH_11 period (Figure 6A).

Furthermore, we focused on the analysis of gene functions that were specifically upregulated during these periods (Figure S3). The genes that were specifically upregulated were all significantly enriched in functional categories related to stress responses. Moreover, genes specifically upregulated only during BBCH_11 and BBCH_13 were mainly enriched in those associated with photosynthesis, whereas genes specifically upregulated only during BBCH_17 were strongly associated with hormonal responses.

We next combined the genes with stage-specific ACRs in their promoter regions and the DEGs identified through RNA-seq. Our analysis revealed that genes with BBCH_11-specific ACRs were most frequently found in cluster 1, although this enrichment was not statistically significant (Table 2). On the other hand, genes with BBCH_13-specific ACRs were significantly overrepresented in cluster 8, which contained genes with high expression at the BBCH_13 stage (Table 2). Similarly, genes with BBCH_17-specific ACRs were predominantly found in cluster 7, which included genes with elevated expression at the BBCH_17 stage (Table 2). These results suggest that changes in gene expression may be influenced by the regulation of ACRs.

We next investigated the cis-regulatory elements (CREs) present within the ACRs located in the promoter regions of genes from clusters 1 and 7 by HOMER. Several motifs associated with abiotic stress and leaf senescence were notably enriched in both clusters (Figure S4). For example, AP2/EREBP family transcription factors, which are known to regulate leaf senescence, were highly enriched in the BBCH_11-specific ACRs of genes in cluster 1 (Figure S4) [33]. Additionally, members of this family are implicated in maize’s response to water-logging stress [34]. *AtERF1* transcription factors were also notably enriched at this stage, and *AtERF1* is known to play a role in JA/ET-regulated defense responses, enhancing resistance to *Botrytis cinerea* [35].

Additionally, several stress-related transcription factors, including *MYB58*, *PIF4*, and *AtMYB93* from the MYB family, were prominently identified in BBCH_17-specific ACRs in cluster 7 genes (Figure S4). *MYB58* is known to regulate lignin biosynthesis by directly binding to AC elements in *Arabidopsis* [36]. *PIF4*, which enhances leaf senescence by promoting key senescence-associated genes (SAGs) like *ABI5*, *EEL*, *EIN3*, and *SGR1*, also acts downstream of *PHOT1* to modulate phototropism in *Arabidopsis* [37,38]. Furthermore, *MYB58* and *AtMYB93*, key members of the MYB-type transcription factor family, are involved in regulating suberin in the root endodermis under non-stress conditions, contributing to plant protection against a variety of stressors [39].

More than 50% of the genes, either in cluster 1 with BBCH_11-specific ACRs or in cluster 7 with BBCH_17-specific ACRs, have motifs of the transcription factors described above in the promoter regions. These findings suggest that the identified transcription factors govern a significant number of genes and may be pivotal in regulating maize leaf development.

To further investigate the genes associated with common peaks, we integrated the DPI-related genes from ATAC-seq with the DEGs identified through RNA-seq (Figure 6B). We found that a total of 93, 185, and 64 upregulated genes with increased ACRs were identified in the BBCH_11 vs. BBCH_13, BBCH_11 vs. BBCH_17, and BBCH_13 vs. BBCH_17 comparisons, respectively. Conversely, 84, 143, and 56 downregulated genes with decreased ACRs were observed in these same comparisons. These results suggest that ACRs may play a critical role in modulating gene expression.

To further investigate the functions of these genes, we performed GO analysis (Figure S5). For the upregulated genes with increased ACRs, the GO terms enriched in the BBCH_11 vs. BBCH_13 comparison were primarily related to stress responses and hormone regulation. In contrast, the BBCH_11 vs. BBCH_17 and BBCH_13 vs. BBCH_17 comparisons revealed significant enrichment in leaf-related processes, such as response to light, photosynthesis, and leaf morphogenesis. For the downregulated genes with decreased ACRs, only a limited number of GO terms were significantly enriched.

Additionally, for the BBCH_11 vs. BBCH_13, BBCH_11 vs. BBCH_17, and BBCH_13 vs. BBCH_17 comparisons, we identified 15, 7, and 0 upregulated ACR-related genes and 11, 2, and 0 downregulated ACR-related genes, respectively, that showed opposite regulation in RNA-seq data (Figure 6B). To investigate whether differentially altered ACRs were enriched for specific transcriptional repressors, we performed HOMER analysis for the BBCH_11 vs. BBCH_13 comparison (Table S7). Several known transcriptional repression factors were identified, including *IbMYB44*, a well-established negative regulator of secondary metabolite pathways, which was significantly enriched in the BBCH_11 vs. BBCH_13 comparison (Table S7). Additionally, *BOS1* was notably enriched in this comparison (Table S7). These results suggest that the discrepancies between the ATAC-seq and RNA-seq data might be attributed to the binding of repressor factors to the promoter regions of certain genes.

## 4. Discussion

Understanding the developmental process of maize leaves is essential for elucidating plant growth mechanisms and enhancing crop yields. As the primary photosynthetic organ, maize leaves directly influence crop productivity through their efficiency in photosynthesis [40]. Chromatin accessibility is a critical factor in the regulation of gene expression, and investigating the dynamics of chromatin openness can provide insights into the regulatory mechanisms that govern genes at various developmental stages [41]. In this study, we employed both ATAC-seq and RNA-seq techniques to analyze maize B73 leaves at the BBCH_11, BBCH_13, and BBCH_17 developmental stages, focusing on the dynamic changes in chromatin accessibility and their relationship with gene expression. This research not only offers novel perspectives on the epigenetic regulation during maize leaf development but also establishes a foundation for crop genetic improvement and functional genomics, bearing significant scientific and practical implications.

Through the genome-wide profiling of chromatin accessibility in maize leaf tissue at various developmental stages using ATAC-seq, we observed an inverse correlation between leaf age and the number of accessible chromatin peaks. Specifically, chromatin accessibility was highest during the early BBCH_11 stage of leaf development, suggesting a more complex regulatory mechanism governing the proliferation and differentiation of younger leaves compared to older ones. Numerous species, including humans, plants, and mice, have undergone similar chromatin accessibility assessments [42,43,44], providing important information for furthering genomic research. Previous hypotheses have suggested that the eukaryotic nucleosomes’ hierarchical structure and compaction can divide the genome into active areas—like enhancers and promoters—and inactive regions that are not involved in transcription [45]. Numerous investigations, including this one, have demonstrated that the placement of accessible chromatin segments (ACRs) is enriched in promoters, which is consistent with these theories [46,47]. Our findings are consistent with earlier research showing that the majority of ACRs were mapped to introns and intergenic regions, with promoters and exons following in roughly equal amounts. Despite differences in genome size and annotation among species, the distribution of chromatin accessibility peaks exhibits remarkable similarity [48,49,50]. This conservation across species or tissues suggests a fundamental regulatory mechanism. The ACR length and number varied with the stage and were associated with gene expression levels in our study. Interestingly, ACRs in intergenic areas were the shortest, while those in 3’ UTR regions were noticeably longer than those in other regions. Furthermore, the expression levels of genes with longer ACRs or several ACRs in their proximal promoter regions were generally greater. These findings, which are corroborated by earlier studies, imply that chromatin accessibility at promoters might affect the levels of gene expression [51,52]. These results collectively suggest that chromatin accessibility regulates gene expression in a conserved fashion across animals.

Throughout developmental phases, ACRs show significant diversity across many tissues and cell types [53,54,55]. For example, more OCRs have been found in pistils than in anthers, despite the fact that there is a considerable overlap [56]. We discovered both tissue-specific and shared ACRs during maize leaf development in the current investigation. Several classical leaf signaling pathways and leaf-related GO keywords showed enrichment in genes linked to stage-specific or common ACRs. Interestingly, several genes showed variable peak intensities (DPIs) or dynamic ACRs at various phases, demonstrating the usefulness of ATAC-seq in efficiently examining the impact of ACRs on leaf development.

In order to comprehend the mechanisms behind the spatiotemporal expression of genes during maize leaf development, we also investigated the connections between transcription factors (TFs) and ACRs by combining transcriptome data and chromatin accessibility. It is interesting to note that for several genes annotated with DPIs, our ATAC-seq and RNA-seq analyses showed conflicting expression trends. This prompted us to speculate that possible transcriptional repressors may be important regulators of leaf growth.

Motif analysis identified enriched TFs, particularly in the BBCH_11 vs. BBCH_13 comparison. Among these, *IbMYB44*, a well-established negative regulator of secondary metabolite pathways, was significantly enriched (Table S7). Consistent with its role in sweet potato, where *IbMYB44* represses anthocyanin biosynthesis [57], this function appears to be conserved across various plant species. In Arabidopsis and other plants, *MYB44* has been extensively characterized as a transcriptional repressor, particularly under stress conditions, where it suppresses the expression of ABA-responsive genes [58,59]. Moreover, RNA-seq analyses have revealed that *BnaMYB44* homologs act as repressors in the biosynthesis of phenylpropanoids and flavonoids, further supporting its conserved role in regulating these metabolic pathways [60].

In the BBCH_11 vs. BBCH_13 comparison, *BOS1* was also significantly enriched (Table S7). An *R2R3MYB* transcription factor, which is encoded by *BOS1*, has the ability to either activate or inhibit transcription [61]. Reactive oxygen species (ROS) intermediates are probably how the *BOS1* gene mediates reactions to biotic and abiotic stress signals [62]. *BOS1* and other *R2R3MYB* proteins have been linked to the control of gene expression in relation to stress reactions, cell death, and a number of metabolic processes, including tryptophan production and the phenylpropanoid pathway. Therefore, we hypothesize that the binding of repressors to the promoter regions of specific genes may be the cause of the contradicting tendencies seen between ATAC-seq and RNA-seq data.

## 5. Conclusions

This study’s findings show that during maize leaf development, there are dynamic changes in chromatin accessibility. We discovered that changes in accessible chromatin regions (ACRs) had a major impact on the expression of associated genes by combining ATAC-seq and RNA-seq investigations. A number of important cis-regulatory elements (CREs) and putative transcriptional repressors implicated in the control of leaf development were also found in this investigation. These results offer insightful information and lay the groundwork for further studies on the development of maize leaves.

## Figures and Tables

**Table 1:** Results of the differential peak intensity (DPI) analysis for common ACRs.

Group	Up	Down	Total
BBCH_11 vs. BBCH_13	898	1721	2619
BBCH_11 vs. BBCH_17	610	1165	1775
BBCH_13 vs. BBCH_17	587	127	714

**Table 2:** Statistical significance of the overlap between stage-specific ACR-related genes and differentially expressed genes in eight clusters.

	Genes Associated with Specific ACRs		
	BBCH_11(4158)	BBCH_13(3)	BBCH_17(33)
C1	7%(1)	0%	0%
C2	1%(1)	0%	0%
C3	5%(1)	0%	0%
C4	0%	0%	3%(0.774)
C5	6%(0.999)	0%	0%
C6	2%(1)	0%	0%
C7	4%(1)	0%	91%(0.01)
C8	2%(1)	67%(0.04)	0%

Note: The enrichment significance, as determined by Fisher’s exact test, is presented in parentheses. The percentages represent genes that exhibit stage-specific ACRs distributed across C1 to C8.

## Data Availability

The data supporting the findings of this study are included within the article and its Supplementary Files. The original contributions are available in the article and Supplementary Materials. For further inquiries, please contact the corresponding author.

## Supplementary Materials

The following supporting information can be downloaded at https://www.mdpi.com/article/10.3390/genes15121630/s1: Figure S1: Correlation analysis between samples in RNA-seq experiments; Figure S2: Landscape of stage-specific ACR-associated genes; Figure S3: Functional analysis of DEGs in the C1 to C8 groups; Figure S4: Enrichment in tissue-specific open chromatin of recognized transcription factor (TF) binding motifs; Figure S5: Functional analysis of DEGs overlapping with DPI-related genes. Table S1: The samples’ ATAC-seq sequencing statistics. Table S2: Details about the RNA-seq libraries are shown. Table S3: Stage-specific ACRs across several phases were analyzed using GO. Results from the GO analyses of stage-specific ACRs at the BBCH_11 stage. Table S4: GO analyses of common ACRs with DPIs. Findings from the GO analysis of typical ACRs with DPIs found in the BBCH_11 versus BBCH_13. Table S5: GO analyses of typical ACRs with DPIs are shown. Findings from the GO analysis of typical ACRs with DPIs found in the BBCH_11 versus BBCH_17. Table S6: GO analysis of common ACRs with DPIs identified from the BBCH_13 vs. BBCH_17 comparison. Table S7: Enrichment of known transcription factor (TF) motifs in common ACRs with DPIs from the BBCH_11 vs. BBCH_13 comparison.
