# Supplementary figures and images for: Dynamic Chromatin Accessibility and Gene Expression Regulation During Maize Leaf Development
Source: Genes (Basel). 2024 Dec 20;15(12):1630. doi: 10.3390/genes15121630 (PMC11675475; doi:10.3390/genes15121630)

Fig. S3

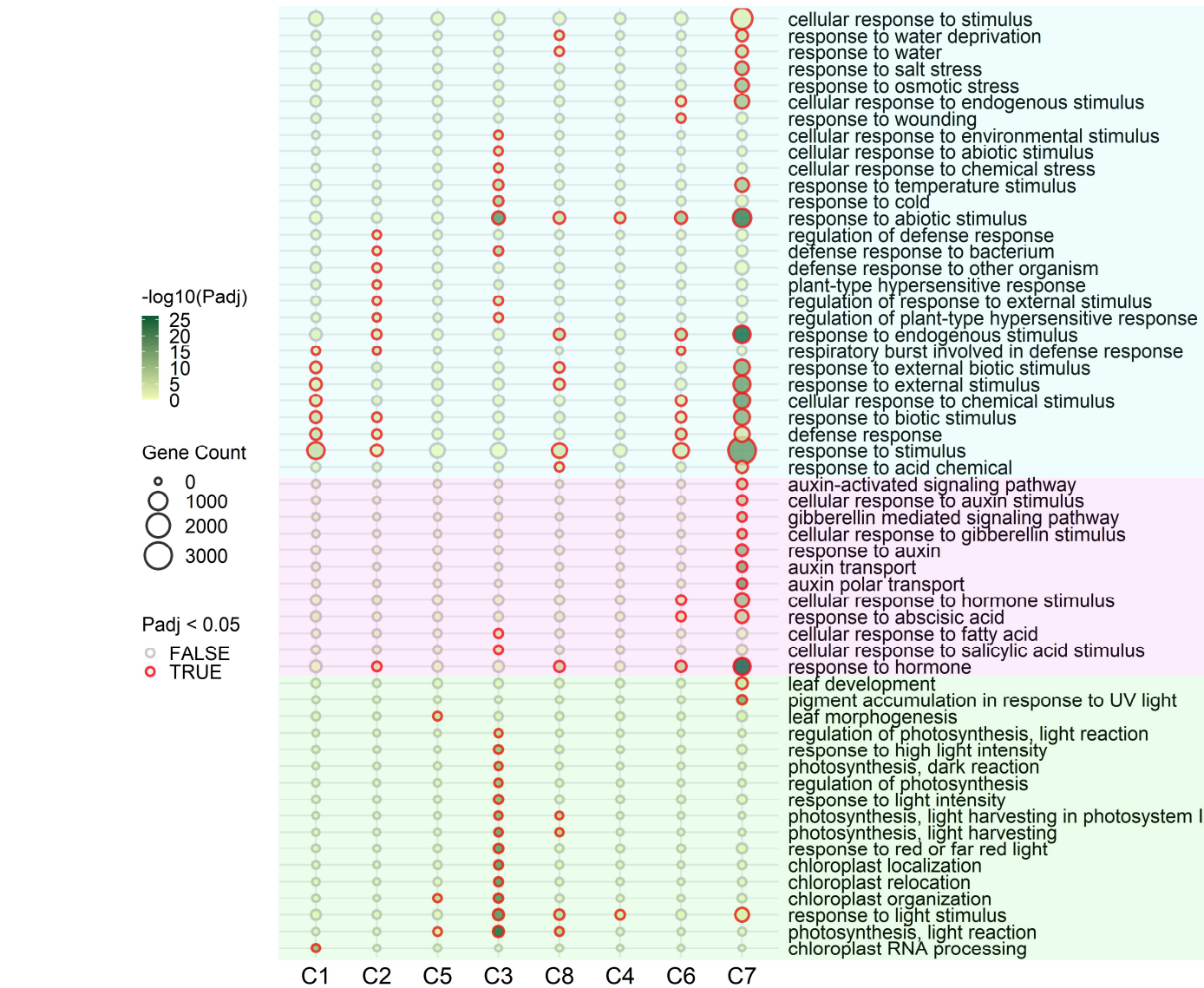

Supplement: Supplementary file 1 [file genes-15-01630-s001.zip › fig.S3.pdf]

Fig. S4

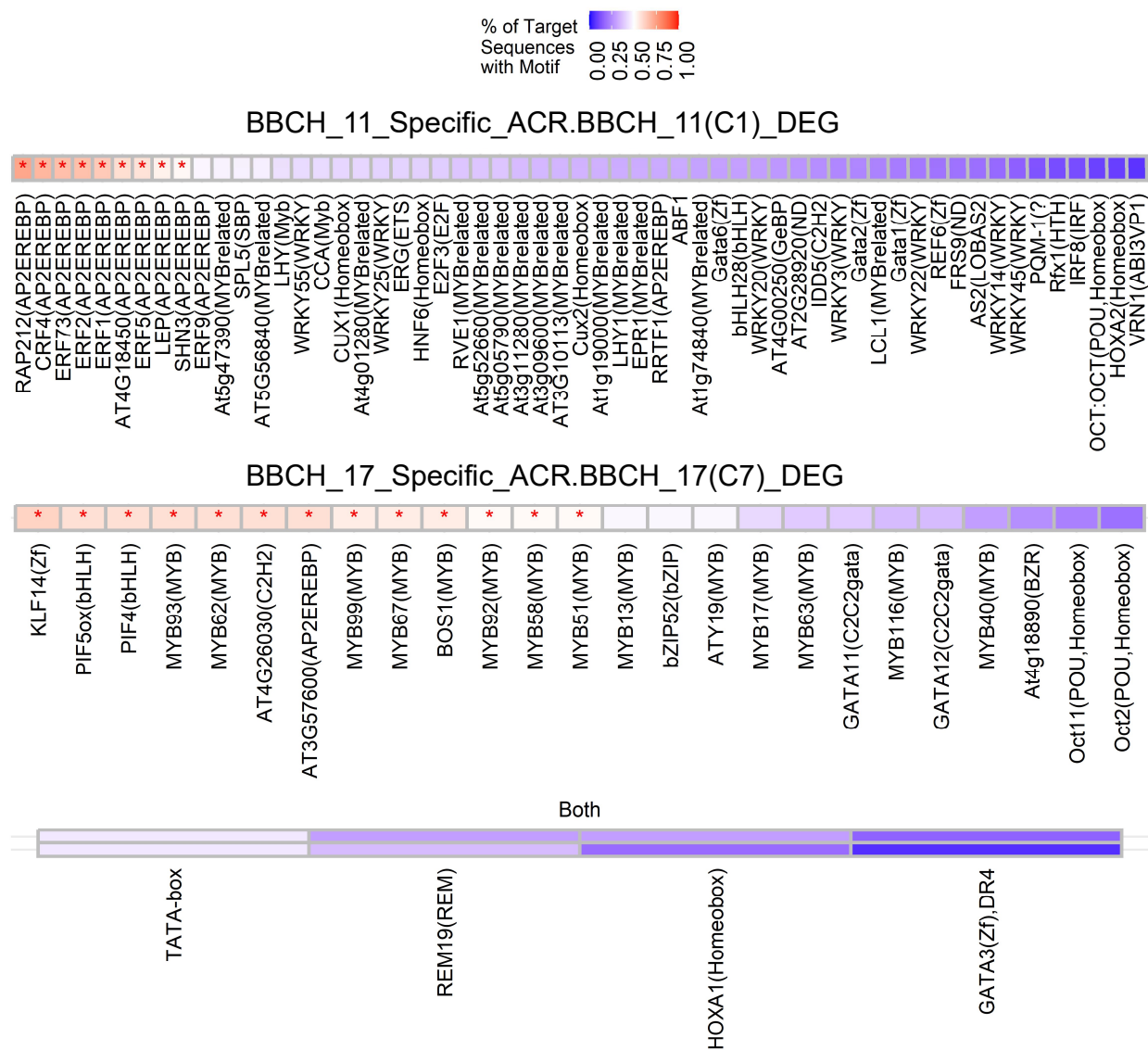

Supplement: Supplementary file 1 [file genes-15-01630-s001.zip › fig.S4.pdf]

Fig. S5

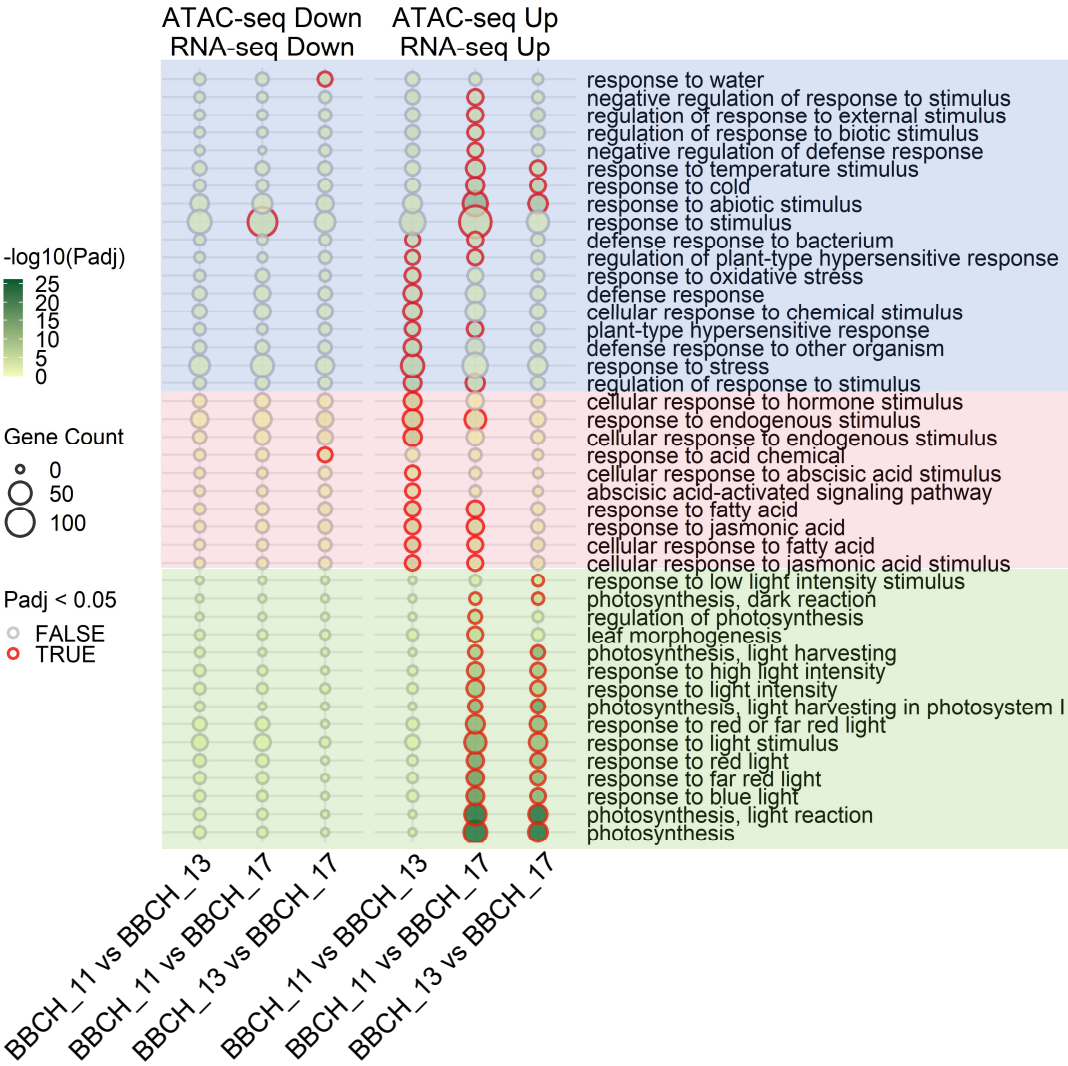

Supplement: Supplementary file 1 [file genes-15-01630-s001.zip › fig.S5.pdf]

Fig. S1

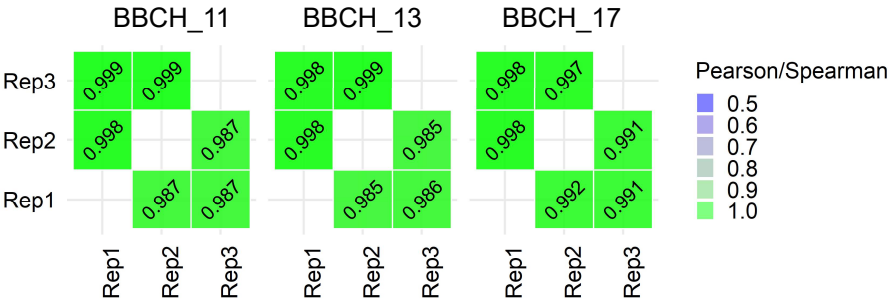

Supplement: Supplementary file 1 [file genes-15-01630-s001.zip › fig.S1.pdf]

Fig. S2

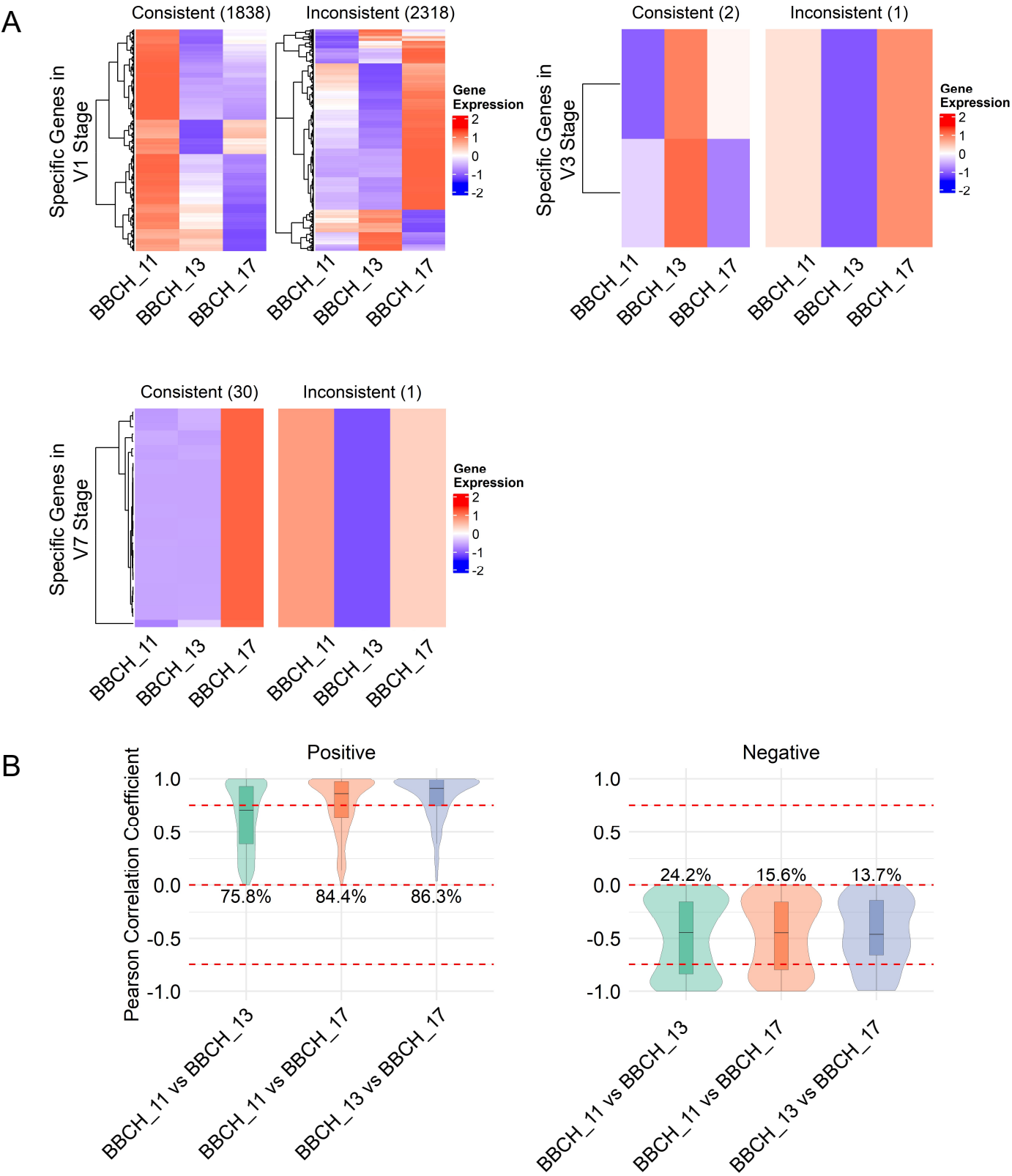

Supplement: Supplementary file 1 [file genes-15-01630-s001.zip › fig.S2.pdf]
